# Falls and Fall-Related Injuries Among US Adults Aged 65 or Older With Chronic Kidney Disease

**DOI:** 10.5888/pcd15.170518

**Published:** 2018-06-21

**Authors:** Brandon M. Kistler, Jagdish Khubchandani, Gina Jakubowicz, Kenneth Wilund, Jacob Sosnoff

**Affiliations:** 1 Department of Nutrition and Health Science, Ball State University, Muncie, Indiana; 2 Department of Kinesiology and Community Health, University of Illinois at Urbana-Champaign, Urbana, Illinois

## Abstract

**Introduction:**

Falls are among the leading causes of injury and death among adults aged 65 or older. People with chronic kidney disease (CKD) are at increased risk of falling and of having a serious injury from falls. However, information is limited about risk factors for falls and fall-related injuries among people with CKD.

**Methods:**

We performed a secondary analysis of 157,753 adults (6.1% with CKD) aged 65 or older surveyed in the 2014 Behavioral Risk Factor Surveillance System.

**Results:**

People with CKD were at increased risk of falls (odds ratio [OR] = 1.81; 95% confidence interval [CI], 1.63–2.01) and fall-related injuries (OR = 1.50; 95% CI, 1.27–1.78) even after adjusting for differences in demographic characteristics, health conditions, and lifestyle factors (*P* < .05 for all). Among people with CKD, women, people diagnosed with diabetes, diabetes duration, and arthritis were all significant predictors of falls and fall-related injuries (*P* < .05 for all). Lifestyle factors, such as engaging in recent exercise (adjusted odds ratio [AOR] = 0.68; 95% CI, 0.56–0.81) and limited physical function (assessed as difficulty in climbing stairs) (AOR = 2.84; 95% CI, 2.30–3.44), were most closely associated with falls and fall-related injuries.

**Conclusion:**

Adults aged 65 or older with CKD were at increased risk of falling and of suffering an injury as a result of a fall compared with adults in the same age range without CKD. Potentially modifiable factors such as physical function and recent exercise were most closely related to reduced risk for falls and fall-related injuries and may be an appropriate target for fall prevention and rehabilitation programs in people with CKD.

## Introduction

Falls are among the leading causes of injury and death among US adults aged 65 or older (1). Nearly one-third of adults in this age group report a fall every year (2), and the annual cost of falls in the United States is approximately $31 billion (3). Numerous risk factors for falls have been identified, including frailty and chronic diseases (4).

Chronic kidney disease (CKD) is common among adults aged 65 and older with an estimated prevalence of 14.8% in the US population (5). People with CKD in this age group have a greater risk of falling than those without CKD (4). Furthermore, poor kidney function is a risk factor for falling (6) and for poor outcomes from falls, such as fractures (7). People with CKD may be more likely to fall and to experience a serious injury from falls; however, information is limited on the prevalence and predictors of falls in adults aged 65 or older with CKD (8).

Previous studies have identified age, sex, frailty, and diabetes as risk factors for unintentional falls (8). However, published data have limitations. For example, previous studies primarily used convenience samples from small clinics or health care facilities, assessed falls without exploring fall-related injuries, and often did not focus on older adults. Thus, these studies provided limited assessments of health, lifestyle, and demographic factors that may influence prevalence of falls or CKD. The limited samples in these previous studies, although providing valuable information, have led to conflicting results in relation to many risk factors. For example, some studies of people with CKD found that men were at increased risk of falling (9), whereas others found that women were more likely to fall (10,11). Thus, the purpose of this study was to assess the prevalence of CKD and falls in a large national sample of US adults aged 65 or older and to explore the association between falls, CKD, health risk factors, and demographic characteristics.

## Methods

### Study design and participants

We conducted a secondary analysis of data from the 2014 Behavioral Risk Factor Surveillance System (BRFSS). BRFSS is conducted annually by the Centers for Disease Control and Prevention (CDC) to measure behavioral and health risk factors and diseases in US adults. BRFSS is a telephone survey that uses random-digit dialing to randomly select civilian noninstitutionalized adults aged 18 or older. In 2014, BRFSS data were collected from adults across all 50 states and the District of Columbia. BRFSS uses a complex multistage sampling procedure and design weights to adjust for the unequal probability of being selected, for noncoverage, and for nonresponses. This is to ensure the creation of equal population estimates for each geographic region. The combined landline and cellular telephone median weighted response rate was 47.0% (landline telephones, 48.7%; cellular telephones, 40.5%). The BRFSS questionnaire consists of 3 parts: 1) core questions, which are a standard set of questions that all participating states and territories must administer; 2) optional modules; and 3) state-added questions*,* that is, questions on specific topics that states can choose to include in response to state-specific health concerns. Additional details about BRFSS survey methods, sampling, and response rates are available (12). Because BRFSS is approved by CDC’s institutional review board (IRB) and because our study used de-identified publicly available data, did not recruit human subjects, and had no direct contact with study participants, no additional approval was required from the authors’ IRBs.

### Measures

**Demographic characteristics and chronic kidney disease.** We included all adults aged 65 or older who participated in the 2014 BRFSS (N = 157,753). BRFSS also collected data on participants’ sex, race, marital status, employment, and education through structured and closed-format questions; we used these demographic variables for our analysis. BRFSS 2014 asked respondents about history of diagnosis of various chronic conditions including CKD with response options of yes, no, or don’t know. The CKD diagnosis question was, “Has a doctor, nurse, or other health professional ever told you that you have kidney disease (excluding kidney stone, bladder infection, or incontinence)?” (12). Respondents were categorized into 2 groups based on a history of CKD to assess differences in demographic characteristics and lifestyle factors (ie, CKD group vs non-CKD group).

**Health, lifestyle, and disease conditions. **Study participants were asked if they had ever been diagnosed with arthritis, diabetes, or cancer (with response options of yes, no, or don’t know). Response options to questions on current smoking, heavy drinking, difficulty walking, and health coverage were yes or no. Body mass index (BMI) (weight in kg/height in m^2^) was computed on the basis of self-reported height and weight. A single item assessed participants’ current general health (with response options of excellent, very good, good, fair, and poor) (12). For prevalence of falls and fall-related injuries, the 2014 BRFSS asked 2 questions: 1) In the past 12 months, how many times have you fallen and 2) how many of these falls caused an injury. The responses were categorized as no for 0 events and yes for 1 or more falls or fall-related injury events (12). CKD and non-CKD groups were compared for differences in these health and disease variables.

### Data analysis

We first computed descriptive statistics (eg, percentages, frequencies) for all study variables and measures (ie, demographics, lifestyle behaviors, and chronic conditions). Using χ^2 ^tests we explored the differences in these variables between respondents with a history of CKD versus those without. Second, we used the binary variable history of CKD (yes vs no) as an independent variable to predict the odds of falls and fall-related injuries. In multivariate logistic regression analysis, falls and fall-related injuries were used as an outcome, with CKD as a predictor, and we computed adjusted odds for falls and fall-related injuries after adjusting for demographic characteristics of study participants, their lifestyle and health behaviors, and history of comorbid conditions that may be associated with falls or CKD. All analyses were performed by using the complex sample survey data analysis procedures in SPSS version 24 (IBM Corp). Statistical significance was set a priori at *P* < .05.

## Results

Most study participants were white (79%), female (56%), retired (72%), and married or living with a partner (56%). Slightly more than a quarter of the participants were obese (28%), reported difficulty in walking (27%), and had poor or fair health (26%). In relation to falls, almost a third of participants (29%) had a fall in the past 12 months, and 10% had a serious injury resulting from the fall. Less than a tenth of participants reported a history of CKD (6.1%). A comparison of adults aged 65 or older with and without CKD revealed differences based on demographic characteristics, lifestyle behaviors, and comorbid conditions between adults with and without CKD (Table 1).

**Table 1:** Participant (N = 157,753) Characteristics, Study of Falls and Fall-Related Injuries Among US Adults Aged 65 or Older With Chronic Kidney Disease (CKD), Behavioral Risk Factor Surveillance System, 2014^a^

Variable	Total, N (%)	CKD, N (%), 9,116	No CKD, N (%), 147,893
**Demographic Characteristics**			
**Sex**			
Male	59,746 (44)	3,547 (45)	55,924 (44)
Female	98,007 (56)	5,569 (55)	91,969 (56)
**Race^b^**			
White	132,276 (79)	7,431 (75)	124,845 (79)
African-American	9,323 (9)	699 (12)	8,624 (9)
Other	3,957 (4)	254 (4)	3,703 (3)
Multiracial	2,129 (1)	167 (1)	1,962 (1)
Hispanic	6,583 (8)	406 (8)	6,177 (8)
**Marital status^b^**			
Married/ living with a partner	76,769 (56)	4,065 (52)	72,433 (56)
Separated/divorced	23,031 (14)	1,430 (15)	21,475 (13)
Widowed	49,687 (27)	3,154 (31)	46,236 (26)
Never married	7,212 (4)	419 (3)	6,750 (4)
**Employment^b^**			
Employed for wages	15,544 (10)	501 (5)	15,043 (10)
Self-employed	8,707 (5)	316 (3)	8,391 (5)
Retired	112,707 (72)	6,695 (74)	106,012 (72)
Other (unable to work, out of work, student, homemaker)	18,567 (13)	1,535 (18)	17,032 (13)
**Education^b^**			
≤High school graduate	65,267 (48)	4,005 (50)	61,262 (48)
>High school but <college graduate	40,050 (28)	2,445 (30)	37,605 (28)
≥College graduate	50,533 (23)	2,601 (19)	47,932 (24)
**Physical Function, Health, and Lifestyle Factors**			
**General health^b^**			
Excellent	20,506 (12)	335 (4)	20,143 (13)
Very good	47,064 (28)	1,353 (14)	45,587 (29)
Good	51,967 (33)	2,835 (31)	48,881 (33)
Fair	26,449 (18)	2,654 (31)	23,609 (17)
Poor	10,972 (8)	1,895 (20)	8,940 (7)
**Access to health care**			
Yes	154,846 (98)	8,976 (98)	145,870 (99)
No	1,789 (2)	124 (2)	1,665 (1)
**Current smoker**	12,736 (9)	650 (7)	12,086 (9)
**Heavy drinker (men >2 drinks/day; women >1 drink/day)**	5,900 (4)	219 (3)	5,681 (4)
**Difficulty walking/climbing stairs^b^**	40,615 (27)	4,471 (52)	36,198 (26)
**Engaged in any exercise** **in past month^b^**	108,953 (69)	5,189 (56)	103,764 (69)
**Obese (BMI ≥30)^b^**	39,566 (28)	3,162 (38)	36,404 (27)
**Chronic conditions in addition to CKD (ever diagnosed) **			
Diabetes^b^	32,429 (23)	3,591 (43)	28,838 (22)
Cancer	27,133 (17)	2,441 (29)	24,692 (16)
Arthritis^b^	84,017 (53)	6,344 (72)	77,673 (52)
**Had ≥1 falls in past year^b^**	43,885 (29)	3,529 (41)	40,356 (28)
**Had fall-related injury in past year^b^**	16,062 (10)	1,566 (16)	14,496 (10)

a Not all BRFSS respondents answered the question about CKD. Percentages may not total 100% because of missing values. Percentages are rounded to the nearest whole number.

b Significant differences between groups (*P* < .05).

In a logistic regression analysis (Table 2), we found that people with CKD were more likely to report having falls (OR = 1.81; 95% CI, 1.63–2.01), even after adjusting for demographics, lifestyle behaviors, and comorbid conditions (adjusted odds ratio [AOR] = 1.26; 95% CI, 1.13–1.47). Moreover, 37.4% of those who fell had a fall-related injury, with injuries occurring more frequently among people with CKD (OR = 1.50; 95% CI, 1.27–1.78), even after adjusting for demographic characteristics, lifestyle behaviors, and comorbid conditions (AOR = 1.23; 95% CI, 1.04–1.40).

**Table 2:** Probability of Falls and Fall-Related Injuries, Among US Adults (N = 157,753) Aged 65 or Older With Chronic Kidney Disease (CKD) (N = 9,116) and Without CKD (N = 147,893), Behavioral Risk Factor Surveillance System, 2014^a^

Predictors	OR (95% CI) Falls	OR (95% CI) Fall-Related Injury
**Model 1. **Compares CKD group vs non-CKD group	1.81 (1.63–2.01)^b^	1.50 (1.27–1.78)^b^
**Model 2. **Comparison in Model 1 adjusted for demographic characteristics from Table 1	1.75 (1.58–1.94)^b^	1.46 (1.24–1.72)^b^
**Model 3. **Comparison in Model 1 adjusted for physical function, health, and lifestyle factors from Table 1	1.36 (1.21–1.53)^c^	1.26 (1.08–1.44)^c^
**Model 4. **Comparison in Model 1 adjusted for chronic conditions from Table 1	1.53 (1.38–1.70)^b^	1.42 (1.20–1.69)^b^
**Model 5. **Comparison in Model 1 adjusted for demographic characteristics and physical function, health, and lifestyle characteristics from Table 1	1.32 (1.18–1.48)^c^	1.25 (1.06–1.49)^c^
**Model 6. **Comparison in Model 1 adjusted for demographic, physical function, health/lifestyle characteristics and chronic conditions	1.26 (1.13–1.47)^c^	1.23 (1.04–1.40)^c^

Abbreviations: CI, confidence interval; OR, odds ratio.

a Not all participants responded to the question about CKD.

b Indicates *P* < .001.

c Indicates *P* < .01.

Among patients with CKD, men were significantly less likely than women to fall (AOR = 0.79; 95% CI, 0.65–0.93) and have fall-related injuries (AOR = 0.59; 95% CI, 0.44–0.80), after adjusting for race and age (Table 3). Having a diagnosis of diabetes was associated with an increased likelihood of falling (AOR = 1.25; 95% CI, 1.02–1.53), and the length of time since diabetes diagnosis was associated with both falls and fall-related injuries. People with CKD and arthritis were more likely to fall (AOR = 1.79; 95% CI, 1.46–2.20) and have fall-related injuries (AOR = 1.54; 95% CI, 1.06–2.24), whereas people with cancer were more likely to have a fall-related injury only (AOR = 1.50; 95% CI, 1.04–2.15). Lifestyle factors such as engaging in recent exercise (AOR = 0.68; 95% CI, 0.56–0.81) and limited physical function (assessed as difficulty in climbing stairs) (AOR = 2.84; 95% CI, 2.30–3.44) were most strongly associated with both falls and fall-related injuries.

**Table 3:** Predictors of Falls and Fall-Related Injuries in People With Chronic Kidney Disease, Behavioral Risk Factor Surveillance System, 2014

Predictors	AOR (95% CI)^a^ Falls	AOR (95%CI)^a^ Fall-Related Injury
**Men versus women**	0.79 (0.65–0.93)^b^	0.59 (0.44–0.80)
**Married/ living with a partner versus other^c^**	0.96 (0.78–1.17)	0.90 (0.69–1.08)
**Education**		
**≤**High school graduate	1 [Reference]	1 [Reference]
>High school but <college graduate	1.37 (0.98–1.76)	1.17 (0.81–1.72)
≥College graduate	1.01 (0.83–1.22)	0.72 (0.54–0.90)^b^
**Any exercise last month, yes versus no**	0.68 (0.56–0.81)^b^	0.70 (0.60–0.93)^b^
**Difficulty climbing/walking stairs, yes versus no**	2.84 (2.30–3.44)^b^	1.70 (1.27–2.30)^b^
**Obese or overweight versus normal weight^d^**	0.87 (0.67–1.12)	0.86 (0.60–1.27)
**Heavy drinker** **versus others^e^**	1.54 (0.89–2.98)	1.19 (0.93–2.07)
**Current smoker versus others^f^**	1.21 (0.85–1.71)	1.08 (0.90–1.44)
**Has diabetes versus does not have diabetes**	1.25 (1.02–1.53)^b^	1.07 (0.81–1.44)
**Diabetes diagnosed** **≤64 y versus diagnosed ≥65 y**	1.45 (1.04–2.02)^b^	1.62 (1.08–2.53)^b^
**Arthritis history, yes versus no**	1.79 (1.46–2.20)^b^	1.54 (1.06–2.24)^b^
**Cancer history, yes versus no**	1.03 (0.81–1.30)	1.50 (1.04–2.15)^b^

Abbreviations: AOR, adjusted odds ratio; CI, confidence interval.

a Indicates adjustments made for race and age. The outcome is falls and fall-related injuries in the past 12 months (yes vs no).

b Indicates *P* < .01.

c Includes widowed, divorced, separated, and never married.

d People with BMI ≥25 were categorized as overweight or obese, and people with BMI from 18.5 to <25 were categorized as normal weight.

e Men who regularly consume more than 2 drinks per day and women who regularly consume more than 1 drink per day. Others were those who consumed fewer drinks or did not drink alcohol at all.

f Includes nonsmokers and former smokers.

## Discussion

We used secondary data from the 2014 BRFSS to study the relationship between falls in noninstitutionalized adults with and without CKD. After accounting for multiple demographic characteristics, lifestyle factors, and chronic disorders, people with CKD aged 65 and older had a higher prevalence of falls and fall-related injuries than those without CKD. Among people with CKD, multiple lifestyle factors (eg, not currently engaging in physical activity, difficulty climbing stairs) and comorbid conditions (diabetes, diabetes duration, cancer, and arthritis) were found to significantly influence the probability of falls and fall-related injuries. Factors related to exercise and physical function were most closely related to falls and fall-related injuries, suggesting that these may be potential targets of fall-prevention strategies in older adults with CKD.

Our findings are consistent with previous reports that found that patients with CKD (4) and those who have advanced to end-stage kidney disease (ESKD) (10,11,13) are at increased risk of falling. Numerous physiological changes associated with CKD, such as uremic neuropathy and muscle wasting and weakness may explain the increased risk of falling. Furthermore, changes in bone and mineral metabolism leading to weak, brittle bones may lead to an increased propensity for fall-related injuries in people with CKD, especially those with ESKD (12). The increased risk of falls and fall-related injuries is a significant finding because of its strong relationship with poor clinical outcomes (13,14).

Previous studies of people with CKD identified numerous risk factors for falls, including age, sex, body weight, and education (8). However, these studies were primarily of small prospective cohorts and produced conflicting results in relation to some risk factors, such as sex. In our study, we used a large sample of US adults aged 65 and older and found that women with CKD were more likely than men to fall or have a fall-related injury. This is similar to the overall population of adults in the age group from BRFSS in which women were also found to be at greater risk of both falls and fall-related injuries than men (1). Furthermore, we did not find some previously identified demographic factors, such as BMI and education, to be significant predictors of falls in our study sample.

Previous studies also identified diabetes, a leading cause of CKD, as a risk factor for falls among people with CKD (14). Peripheral diabetic neuropathy is a common complication associated with poor glycemic control and can lead to balance and gait impairment, especially in activities such as walking, climbing, and descending stairs. (15). We previously reported greater gait impairments in CKD patients undergoing hemodialysis who also had diabetes compared with those who did not have diabetes (16). Despite these observations, previous studies of fall risk have not accounted for the duration of diabetes. In our study, we found that people with CKD aged 65 or older who were diagnosed with diabetes before age 65, and therefore would have likely lived with diabetes for longer than those diagnosed after 65, were more likely to suffer a fall or fall-related injury. This finding suggests that, similar to the non-CKD populations (17), the physiological changes associated with diabetes that lead to increased injury risk may take time to manifest and that early prevention and management of diabetes may reduce the risk of falls and fall-related injuries in older adults.

Although not as common as diabetes, cancer and arthritis increase fall risk in the general population and are common chronic disorders among people with CKD (4,18,19). In our study, we found that among people aged 65 and older, having cancer and CKD did not increase the risk of falling but did increase the likelihood of suffering a fall-related injury. This increase in injury risk may be due in part to the effect of cancer treatments on bone strength (20).

Factors related to poor health, reduced physical functioning, and chronic diseases had the largest influence on the probability of both falls and fall-related injuries. For example, a diagnosis of arthritis was associated with an increased likelihood of having both a fall and a fall-related injury. Frailty, a condition consisting of fatigue, weakness, and reduced physical activity, is a strong predictor of falls in both the elderly population and in people with CKD who have advanced to ESKD (9). A common strategy to prevent falls is improving strength and balance. We found that difficulty climbing stairs, a task that requires strength and balance (21), was most closely related to falls and fall-related injuries among people with CKD. Furthermore, the absence of exercise in the past month was also a strong predictor of falls and fall-related injuries. Physical function and exercise are potentially modifiable, cost effective, and evidence-based strategies available to enhance mobility. Our study suggests that as in other populations, exercise programs that target strength and balance may be an effective strategy for preventing falls and fall-related injuries among people with CKD, but prospective trials are needed (22).

Our study has several limitations. First, the BRFSS questionnaire relies on self-reported health and lifestyle factors. Self-reporting may have limitations such as recall bias, social desirability, and over- or underestimation of health-related variables. Second, this study was cross-sectional, and we cannot establish cause and effect relationships between variables. Third, BRFSS is a closed-format survey, limiting internal validity. Also, few BRFSS items measured CKD and falls, resulting in improper estimation of the nature and extent of CKD and falls. Finally, CKD is a complex phenomenon with a multitude of influences on disease causation and prognosis. Variables that may influence CKD outcome or progression were not captured (eg, diet and nutritional status). People with CKD may not always be available to answer questionnaires such as BRFSS (eg, because of cognitive disabilities or hospitalization), limiting the external validity of our results and the ability to generalize our findings to all elderly adults with CKD. Despite these limitations, our study has several strengths. To our knowledge, this is the largest survey to examine risk factors for falls and fall-related injuries among people aged 65 and older with CKD. Furthermore, this study analyzed several demographic factors and lifestyle behaviors, which adds to the body of knowledge pertaining to CKD and falls in older adults.

The results of our study show that people with CKD have a higher likelihood of falling and having fall-related injuries. However, potentially modifiable factors such as recent exercise and difficulty climbing stairs were most closely related to falls and fall-related injuries. These findings suggest that among elderly people with CKD, as among other elderly populations at risk for falls, poor physical function and balance may be appropriate targets of multifactorial fall-prevention strategies.

## References

[1] Bergen G , Stevens MR , Burns ER . Falls and fall injuries among adults aged ≥65 years — United States, 2014. MMWR Morb Mortal Wkly Rep 2016;65(37):993–8. 10.15585/mmwr.mm6537a2 27656914

[2] Tinetti ME , Speechley M , Ginter SF . Risk factors for falls among elderly persons living in the community. N Engl J Med 1988;319(26):1701–7. 10.1056/NEJM198812293192604 3205267

[3] Burns ER , Stevens JA , Lee R . The direct costs of fatal and non-fatal falls among older adults — United States. J Safety Res 2016;58:99–103. 10.1016/j.jsr.2016.05.001 27620939 PMC6823838

[4] Paliwal Y , Slattum PW , Ratliff SM . Chronic health conditions as a risk factor for falls among the community-dwelling US older adults: a zero-inflated regression modeling approach. BioMed Res Int 2017;2017:5146378. 10.1155/2017/5146378 28459060 PMC5387801

[5] United States Renal Data System. 2016 USRDS annual data report: Epidemiology of kidney disease in the United States. Bethesda (MD): National Institutes of Health, National Institute of Diabetes and Digestive and Kidney Diseases; 2016.

[6] Dukas LC , Schacht E , Mazor Z , Stähelin HB . A new significant and independent risk factor for falls in elderly men and women: a low creatinine clearance of less than 65 ml/min. Osteoporos Int 2005;16(3):332–8. 10.1007/s00198-004-1690-6 15241585

[7] Dukas L , Schacht E , Stähelin HB . In elderly men and women treated for osteoporosis a low creatinine clearance of <65 ml/min is a risk factor for falls and fractures. Osteoporos Int 2005;16(12):1683–90. 10.1007/s00198-005-1903-7 15933802

[8] López-Soto PJ , De Giorgi A , Senno E , Tiseo R , Ferraresi A , Canella C , Renal disease and accidental falls: a review of published evidence. BMC Nephrol 2015;16(1):176. 10.1186/s12882-015-0173-7 26510510 PMC4625452

[9] Cook WL , Tomlinson G , Donaldson M , Markowitz SN , Naglie G , Sobolev B , Falls and fall-related injuries in older dialysis patients. Clin J Am Soc Nephrol 2006;1(6):1197–204. 10.2215/CJN.01650506 17699348

[10] McAdams-DeMarco MA , Suresh S , Law A , Salter ML , Gimenez LF , Jaar BG , Frailty and falls among adult patients undergoing chronic hemodialysis: a prospective cohort study. BMC Nephrol 2013;14(1):224. 10.1186/1471-2369-14-224 24131569 PMC3852906

[11] Abdel-Rahman EM , Yan G , Turgut F , Balogun RA . Long-term morbidity and mortality related to falls in hemodialysis patients: role of age and gender — a pilot study. Nephron Clin Pract 2011;118(3):c278–84. 10.1159/000322275 21212691

[12] Centers for Disease Control and Prevention. 2014 BRFSS survey data and documentation; 2015. https://www.cdc.gov/brfss/annual_data/annual_2014.html. Accessed May 22, 2017.

[13] Rossier A , Pruijm M , Hannane D , Burnier M , Teta D . Incidence, complications and risk factors for severe falls in patients on maintenance haemodialysis. Nephrol Dial Transplant 2012;27(1):352–7. 10.1093/ndt/gfr326 21652549

[14] Bowling CB , Bromfield SG , Colantonio LD , Gutiérrez OM , Shimbo D , Reynolds K , Association of reduced eGFR and albuminuria with serious fall injuries among older adults. Clin J Am Soc Nephrol 2016;11(7):1236–43. 10.2215/CJN.11111015 27091516 PMC4934847

[15] Brown SJ , Handsaker JC , Bowling FL , Boulton AJM , Reeves ND . Diabetic peripheral neuropathy compromises balance during daily activities. Diabetes Care 2015;38(6):1116–22. 10.2337/dc14-1982 25765355

[16] Shin S , Chung HR , Kistler BM , Fitschen PJ , Wilund KR , Sosnoff JJ . Effect of muscle strength on gait in hemodialysis patients with and without diabetes. Int J Rehabil Res 2014;37(1):29–33. 10.1097/MRR.0b013e3283643d76 23873223

[17] Majumdar SR , Leslie WD , Lix LM , Morin SN , Johansson H , Oden A , Longer duration of diabetes strongly impacts fracture risk assessment: the Manitoba BMD Cohort. J Clin Endocrinol Metab 2016;101(11):4489–96. 10.1210/jc.2016-2569 27603908 PMC5095256

[18] Wong G , Hayen A , Chapman JR , Webster AC , Wang JJ , Mitchell P , Association of CKD and cancer risk in older people. J Am Soc Nephrol 2009;20(6):1341–50. 10.1681/ASN.2008090998 19406977 PMC2689896

[19] Barbour KE , Stevens JA , Helmick CG , Luo YH , Murphy LB , Hootman JM , ; Centers for Disease Control and Prevention (CDC). Falls and fall injuries among adults with arthritis — United States, 2012. MMWR Morb Mortal Wkly Rep 2014;63(17):379–83. 24785984 PMC4584889

[20] Baxter NN , Habermann EB , Tepper JE , Durham SB , Virnig BA . Risk of pelvic fractures in older women following pelvic irradiation. JAMA 2005;294(20):2587–93. 10.1001/jama.294.20.2587 16304072

[21] Bennell K , Dobson F , Hinman R . Measures of physical performance assessments: Self-Paced Walk Test (SPWT), Stair Climb Test (SCT), Six-Minute Walk Test (6MWT), Chair Stand Test (CST), Timed Up & Go (TUG), Sock Test, Lift and Carry Test (LCT), and Car Task. Arthritis Care Res (Hoboken) 2011;63(Suppl 11):S350–70. 10.1002/acr.20538 22588756

[22] El-Khoury F , Cassou B , Charles MA , Dargent-Molina P . The effect of fall prevention exercise programmes on fall induced injuries in community dwelling older adults: systematic review and meta-analysis of randomised controlled trials. BMJ 2013;347:f6234. 24169944 10.1136/bmj.f6234 PMC3812467

